# The complete mitochondrial genome of *Hyporhamphus quoyi* (Beloniformes; Hemiramphidae)

**DOI:** 10.1080/23802359.2018.1532339

**Published:** 2018-10-26

**Authors:** Kehua Zhu, Zhenming Lü, Bingjian Liu, Li Gong, Lihua Jiang, Liqin Liu

**Affiliations:** a National Engineering Laboratory of Marine Germplasm Resources Exploration and Utilization, Zhejiang Ocean University, Zhoushan, China;; b National Engineering Research Center for Facilitated Marine Aquaculture, Marine science and technology college, Zhejiang Ocean University, Zhoushan, China

**Keywords:** Hyporhamphus quoyi, mitogenome, phylogenetic tree

## Abstract

The complete mitogenome of *Hyporhamphus quoyi* was sequenced, which is a closed double-stranded circular molecule of 16,511 bp, and we analyzed the main features in terms of the genome organization, gene arrangement and codon usage. The overall base composition includes A(27.4%), T(26.0%), C(30.4%) and G(16.2%). Moreover, the 13 protein-coding genes (PCGs) encode 3804 amino acids in total, 12 of which use the initiation codon ATG except COI uses GTG, most of them have a complete stop codon, whereas Cytb ends with the incomplete stop codon represented as a single T. The phylogenetic tree based on the neighbor-Joining method was conducted to provide relationship within Beloniformes, based on 12 PCGs encoded by the heavy strand, the NJ tree demonstrated that *H. quoyi* has a closest relationship with *Hyporhamphus intermedius*, which could be a useful basis for propagation of this species.

*Hyporhamphus quoyi*, known from Thailand, the East Indies, Borneo, and the Philippines north to China and Nagasaki, Japan, south to New Guinea and northern half of Australia (Tibbetts and Carseldine 2005), they Inhabits more turbid and estuarine situations than does *Hyporhamphus dussumieri*, often foraging in groups and feeding on zooplankton. As an important group of minor commercial fishes in fishing wharfs of China, we described the characterization of complete mitochondrial genome of *Hyporhamphus quoyi* and explored the phylogenetic relationship within Beloniformes, which were expected to facilitate future studies on taxonomic resolution, population genetic structure and phylogenetic relationships.

Specimens of *H. quoyi* were sampled by commercial bottom trawling in the South China Sea (16°48′42″N; 112°22′25″E) and stored in laboratory of Zhejiang Ocean University with accession number 20150826JDS22.

The complete mitochondrial genome of *H. quoyi* is a closed double-stranded circular molecule of 16,511 nucleotides (GenBank accession no. MH714706), consisting of 13 PCGs, 22 tRNA genes, two rRNA genes, one replication origin and a control region, this feature was similar to the typical mitogenome of other vertebrates (Du et al. 2018; Zhu et al. 2018c). The overall base composition is 27.4%, 26.0%, 30.4% and 16.2% for A, T, C and G, respectively, with a slight AT bias 53.4%. The 13 PCGs genes encode 3804 amino acids in total, similar to the typical mitogenome of vertebrates, all the PCGs use the initiation codon ATG except COI use GTG (Miya et al. 2001; Zhu et al. 2018a). Most of them have TAA or TAG as the stop codon, three PCGs (COII, ND4 and ND6) use AGA as the stop codon, while one PCG uses an incomplete stop codon T, these incomplete termination codons were presumably completed as TAA via post-transcriptional polyadenylation (Ojala et al. 1981). The 12S and 16S are 945 bp and 1680 bp, respectively. The tRNA genes were generated using the program tRNAs-can-SE (Lowe and Eddy 1997), all of them can fold into a typical cloverleaf structure except tRNA-Ser (AGY), which lacks a dihydrouridine arm. As in other vertebrates, two non-coding regions are found in the *H. quoyi* mitogenome (Zhu et al. 2018b), the OL is located in a cluster of five tRNA genes (WANCY), which has the potential to fold into a stable stem-loop secondary structure, with a stem formed by 13 paired nucleotides and a loop of 12 nucleotides; The CR, with 858 bp, is located between tRNA-Pro and tRNA-Phe, and the core sequence of the terminal-associated sequence (ACATATATG) was identified in the CR of *H. quoyi*, which is identical to that in other teleostean mitogenomes (Zhang et al. 2013).

To explore the phylogenetic position of this *H. quoyi*, we used MEGA5 (Tamura et al. 2011) to construct a phylogenetic tree based on the NJ analysis of 12 PCGs encoded by the heavy strand of 18 Beloniformes species. the result of the present study supports *H. quoyi* has a closest relationship with *Hyporhamphus intermedius* (Figure 1), which would contribute to the understanding of the phylogeny of Hemiramphidae (Meisner and Burns 1997).

**Figure 1.**
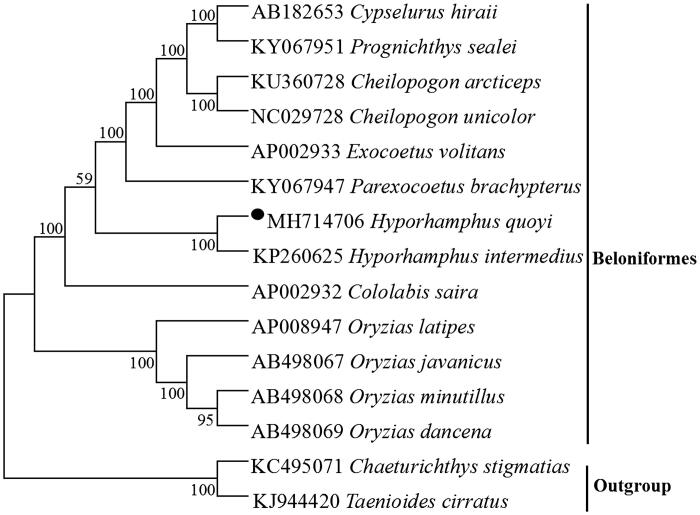
Neighbor joining (NJ) tree of 13 Beloniformes species based on 12 PCGs encoded by the heavy strand. The bootstrap values are based on 1000 resamplings. The number at each node is the bootstrap probability. The number before the species name is the GenBank accession number. The genome sequence in this study is labeled with a black spot.

## References

[CIT0001] Du X, Gong L, Chen W, Liu L, Lü Z 2018 The complete mitochondrial genome of Epinephelus Chlorostigma (Serranidae; Epinephelus) with phylogenetic consideration of Epinephelus. Mitochondrial DNA Part B. 3:209–210. 10.1080/23802359.2018.1436992 PMC7799945 33474120

[CIT0002] Lowe TM, Eddy SR 1997 tRNAscan-SE: a program for improved detection of transfer RNA genes in genomic sequence. Nucleic Acids Res. 25:955–964. 9023104 10.1093/nar/25.5.955 PMC146525

[CIT0003] Meisner AD, Burns JR 1997 Viviparity in the halfbeak genera Dermogenys and Nomorhamphus (Teleostei: Hemiramphidae). J Morphol. 234:295. 29852651 10.1002/(SICI)1097-4687(199712)234:3<295::AID-JMOR7>3.0.CO;2-8

[CIT0004] Miya M, Kawaguchi A, Nishida M 2001 Mitogenomic exploration of higher teleostean phylogenies: a case study for moderate-scale evolutionary genomics with 38 newly determined complete mitochondrial DNA sequences. Mol Biol Evol. 18:1993–2009. 11606696 10.1093/oxfordjournals.molbev.a003741

[CIT0005] Ojala D, Montoya J, Attardi G 1981 TRNA punctuation model of RNA processing in human mitochondrial. Nature. 290:470–474. 7219536 10.1038/290470a0

[CIT0006] Tamura K, Peterson D, Peterson N, Stecher G, Nei M, Kumar S 2011 MEGA5: molecular evolutionary genetics analysis using maximum likelihood, evolutionary distance, and maximum parsimony methods. Mol Biol Evol. 28:2731–2739. 21546353 10.1093/molbev/msr121 PMC3203626

[CIT0007] Tibbetts IR, Carseldine L 2005 Trophic shifts in three subtropical Australian halfbeaks (Teleostei: Hemiramphidae). Mar Freshwater Res. 56:925–932.

[CIT0008] Zhang H, Zhang Y, Zhang X, Song N, Gao T 2013 Special structure of mitochondrial DNA control region and phylogenetic relationship among individuals of the black rockfish, Sebastes schlegelii. Mitochondrial DNA. 24:151–157. 23072475 10.3109/19401736.2012.731401

[CIT0009] Zhu K, Gong L, Jiang L, Liu L, Lü Z, Liu B-j 2018a Phylogenetic analysis of the complete mitochondrial genome of Anguilla japonica (Anguilliformes, Anguillidae). Mitochondrial DNA Part B. 3:536–537. 10.1080/23802359.2018.1467225 PMC7799773 33474232

[CIT0010] Zhu K, Gong L, Lü Z, Liu L, Jiang L, Liu B 2018b The complete mitochondrial genome of *Chaetodon octofasciatus* (Perciformes: Chaetodontidae) and phylogenetic studies of Percoidea. Mitochondrial DNA Part B. 3:531–532. 10.1080/23802359.2018.1467218 PMC7799963 33474230

[CIT0011] Zhu K, Lü Z, Liu L, Gong L, Liu B 2018c The complete mitochondrial genome of *Trachidermus fasciatus* (Scorpaeniformes: Cottidae) and phylogenetic studies of Cottidae. Mitochondrial DNA Part B. 3:301–302. 10.1080/23802359.2018.1445480 PMC7799978 33474152

